# Functions and underlying mechanisms of miR-650 in human cancers

**DOI:** 10.1186/s12935-022-02551-9

**Published:** 2022-03-24

**Authors:** Yuanshuai Su, Qiuxian Zheng, Lingxiao Zhu, Xinyu Gu, Juan Lu, Lanjuan Li

**Affiliations:** State Key Laboratory for Diagnosis and Treatment of Infectious Diseases, Collaborative Innovation Center for Diagnosis and Treatment of Infectious Diseases, The First Affiliated Hospital, College of Medicine, National Clinical Research Center for Infectious Diseases, Zhejiang University, Hangzhou, 310003 China

**Keywords:** MiR-650, Cancer, Expression, Biological roles, Oncogenesis

## Abstract

MicroRNAs (miRNAs) are one type of noncoding RNAs that interfere with mRNA translation to downregulate gene expression, which results in posttranscriptional gene silencing. Over the past two decades, miRNAs have been widely reported to impact the progression of malignant tumours by interfering with cancer initiation and progression; therefore, miRNAs represent potential new diagnostic and therapeutic tools. miR-650 is a newly identified miR, and increasing studies have demonstrated that miR-650 plays critical roles in cancer progression, such as mediating the Wnt signalling pathway/AXIN1 (axis inhibition protein 1) axis in hepatocellular carcinoma. Nevertheless, associations between the expression patterns and molecular mechanisms of miR-650 in cancer have not been comprehensively described. In this article, we review the existing evidence regarding the mechanisms by which miR-650 expression is altered and their relation to cancer. Moreover, the promising clinical application of miR-650 for diagnosis and treatment is highlighted.

## Background

Cancer is currently the major cause of death worldwide. By 2030, the number of cancer deaths is estimated to increase to 13 million [1], which will be an immense burden on the worldwide health system and especially on the health systems in low-income countries [2]. Primary cancer prevention, early detection and timely treatment are the most cost-effective to improve the prognosis and reduce the mortality of patients with cancer. Over the past few decades, great efforts have been made to reveal the molecular mechanisms of tumorigenesis, and its management has been dramatically revolutionized by accurately targeted therapies. For example, directly targeted drugs for KRAS (Kirsten rat sarcoma viral oncogene homologue), one of the most frequently mutated oncogenes, have obtained encouraging results in clinical trials [3]. Moreover, immune therapy aimed at immune checkpoints, such as inhibitors of programmed cell death protein 1 (PD-1), provides huge benefits to cancer patients [4]. Despite great improvements in diagnosis and treatment, the patient mortality associated with tumours are still a severe problem in both sexes. Hence, new molecular agents and strategies with high accuracy need to be identified and applied to the early diagnosis and effective treatment of human cancer.

As a class of endogenous and noncoding RNAs run the length of only 20–24 nt [5], miRNAs play a central role in cell proliferation and migration. By interacting with supplementary series in the 3′-untranslated regions of target mRNAs, miRNAs regulate target genes by repressing translation or degrading target mRNAs at the posttranscriptional level. In just over two decades, more than 2600 miRNAs have been uncovered, and the miR field has expanded considerably [6, 7]. Currently, increasing evidence from in vivo and in vitro experiments has revealed that miRNAs play crucial roles as multifunctional regulators of wide-ranging diseases, particularly in cancer. miRNAs are further categorized into tumour promoters and tumour suppressors [8]. For example, miR-34a, one of the best-investigated members of the miR-34 family, has been shown to play a tumour suppressive role in p53-mediated apoptosis by directly targeting the antiapoptotic protein sirtuin1 [9]. In head and neck cancer, miRNA-196a has been shown to promote cisplatin resistance by targeting (cyclin-dependent kinase inhibitor 1B (CDKN1B) [10]. Insights into the molecular mechanisms of miRNAs in cancer have made miRNAs attractive biomarkers for cancer diagnosis and novel therapeutic approaches.

In recent years, numerous studies have revealed that miR-650 plays vital roles in various tumours. miR-650 is up- or downregulated in various tumour types and can act as a tumour suppressor or oncogene. Moreover, miRNA mimics and molecules targeting miR-650 (anti-miR) have shown tremendous potential in preclinical development. One recent study reported that lncRNA MCTP1 upregulated the expression of miR-650 in endometrial cancer (EC) through the miR-650/SMAD7 axis [11] and then modulated the cell proliferation and invasion, and induced epithelial to mesenchymal transition (EMT) process of EC. Inhibitor of growth 4 (ING4) is a well-known tumour suppressor, and some reports have shown that the ING4 expression level is remarkably downregulated in tumour tissues by miR-650 [12–18]. miR-650 also contributes to drug resistance, such as by upregulating dacarbazine (DTIC) resistance in melanoma through the lncRNA POU3F3/miR-650/MGMT (methylguanine-DNA-methyltransferase) axis [19], which turns miR-650 into a potential target for cancer treatment. In addition, miR-650 can influence other diseases, such as rheumatoid arthritis [20, 21], ulcerative colitis, and cerebral ischaemia/reperfusion injury. In this review, we systematically summarize the expression profiles and functions of miR-650 in various tumours, with special emphasis on its target genes, upstream regulators, and interacting molecules. Furthermore, we summarize the clinical application potential of targeting miR-650 in the oncology field.

## Human studies of miR-650

Initially, miR-650 was identified by Cummins et al. [22] using a miRAGE cloning approach in colorectal cells. Over the next few years, it was quickly recognized that miR-650 is conserved and has extensive functional significance throughout the plant and animal kingdoms. Data from GeneCards (https://www.genecards.org) reveal that miR-650 genes are located in the eukaryotic genome on chromosome 22. Sequence analysis and structural predictions in Das’s research [23] revealed that the miR-650 genes were emerged in multiple copies and overlapped with the leader exons of the immunoglobulin lambda variable 2–8 genes located on chromosome 22 in the same transcription orientation. Notably, the miR-650 gene is the only known miRNA gene that overlaps with immunoglobulin genes [24].

## Expression of miR-650 in cancers

### Hepatocellular carcinoma (HCC)

Hepatocellular carcinoma (HCC), the most frequent primary cancer, is the third major cause of tumor-related deaths [25]. It has the characteristics of a high degree of malignancy, poor treatment response and unfavourable prognosis [26] and represents a heavy public health burden. Multiple risk factors [27–29] involved in the complex process of HCC have been widely reported, among which miR-650 is one of the key molecules. For the past few years, dysregulation of miR-650 in HCC has been widely reported. Han et al. [30] found that miR-650 expression significantly overexpressed in HCC tissues, especially in patients with tumour metastasis. Qin’s et al. [31] research revealed that the expression of Axin, which can inhibit the progression of HCC by targeting miR-650, was weak in HCC. Moreover, the overexpression of miR-650, which can be stimulated with benzo[a]pyrene, promotes the pathological process of fatty liver disease and HCC, as implicated in the work of Ge et al. [32]. Another bioinformatic analysis also demonstrated that the relative quantification of miR-650 in liver tissue was markedly increased in non-alcoholic steatohepatitis (NASH) groups [33]. In addition, elevated miR-650 expression was significantly associated with patient differentiation capability and advanced tumour TNM stage [34]. These findings combined with the above data based on cell lines and clinical samples indicate that miR-650 functions as a tumour promoter in HCC. As discussed above, the progression of HCC is closely related to the expression of miR-650, making it a promising biomarker and therapeutic target for the early prevention and applicable treatment of HCC. Nevertheless, the antitumour effect of miR-650 has rarely been reported in HCC.

### miR-650 and lung cancer (LC)

Approximately 2.2 million cases of lung cancer (LC) were newly increased in 2020. LC causes the most cancer-induced deaths (18.0% of total cancer deaths) [35], of which non-small-cell lung cancer (NSCLC) accounts for the majority [36]. Accumulating studies have indicated that miR-650 acts as a tumour promoter in LC, including NSCLC and lung adenocarcinoma (LAD). In the present study, miR-650 was shown to highly express in NSCLC. Huang et al. [16] found that the expression level of miR-650 was substantially higher in LAD tissue samples than in adjacent normal controls. Moreover, the overexpression of miR-650 was dramatically correlated with the clinical characteristics of LAD patients, such as advanced tumour stage, high incidence of lymph node metastasis, and unfavourable prognosis [16]. Furthermore, downregulation of miR-650 reversed the docetaxel resistance of LAD cells [16]. Another study demonstrated that miR-650 overexpressed in NSCLC, and in vitro experiments indicated that miR-650 promoted the cancer cell proliferation and migration, which resulted in low overall survival date of NSCLC patients [13]. Moreover, a miR-650 inhibitor was shown to attenuate si-MEG3-induced promotion of the LC stem cell-like state, migration and invasion in NSCLC [37]. In spite of great advances in diagnostic and therapeutic methods, the overall survival rate of NSCLC patients is still below 15% [38]. Thus, more efforts are needed to explore new effective strategies for the diagnosis and clinical treatment of LC.

### miR-650 and colorectal cancer (CRC)

According to Global Cancer Statistics, more than 1.9 million new cases of colorectal cancer (CRC) were estimated to occur in 2020, causing 935,000 deaths globally. Tumour metastasis develops in approximately 10% of patients in stage I or II and eventually leads to death within 5 years after exsection [39]. Currently, dysregulation of miR-650 has been researched in CRC, and miR-650 has been reported to act as both a tumour promoter or suppressor; therefore, the role and function of miR-650 in CRC remain controversial. Zhou et al. [39] discovered that the expression of miR-650 in CRC tissues positively correlated with the overall survival of patients. Furthermore, it repressed high-risk non-metastatic CRC progression [39] by inhibiting cell growth and invasion. In contrast, some reports have shown that miR-650 is upregulated and functions as an oncogene in CRC [14, 40–42]. Based on what we already know, there is seemingly no consensus about the expression and function of miR-650 in CRC progression. Thus, more research is needed to comprehensively explore the roles of miR-650 in CRC.

### miR-650 and gastric cancer (GC)

Gastric cancer (GC) was responsible for approximately 769,000 deaths in 2020, ranking fourth in mortality and fifth in incidence [43] globally. Due to the diagnosis of GC at early stages with complications, limited treatment options and poor prognoses, GC remains a great clinical challenge [44]. At present, the standard diagnostic methods for GC patients are gastroscopy and biopsy, but the utility of these methods is limited largely due to the invasiveness of GC and limited medical resources [45]. miR-650, as a new tumour biomarker in GC, has been investigated in recent years. One bioinformatic study based on 180 GC patients and 45 healthy individuals indicated that elevated miR-650 expression levels was significantly correlated with the existence of GC [45] and miRNA-650 are evaluated to be a promising and powerful non-invasive biomarker for the detection of GC. Previously, Zhang et al. [15] reported that overexpression of miR-650 promoted GC tumorigenesis in vivo and GC cell clonogenicity in vivo. Moreover, the overexpression of miR-650 has a positively association with the advancement of GC, as demonstrated in the work of Liu et al. [46] and An et al. [47].

### miR-650 and glioma

Glioma, the most common primary tumour in the brain, accounts for approximately one-third of malignant cancers of the central nervous system [48]. Sun et al. [49] found that miR-650 expression was critically increased in glioma tissues and that was more frequently explored in tumours with a high WHO grade or low Karnofsky performance score (P < 0.001). Another study reported that miR-650 overexpressed in glioma tissues and cell lines. Furthermore, intensive expression was significantly correlated with the advanced tumour stage, lymph node metastasis and poor prognoses in glioma patients [50]. In contrast, Xu et al. [51] found that miR-650 was expressed at a low level in glioma tissues and in vitro cell lines.

### miR-650 and melanoma

Cutaneous melanoma is a common tumour derived from the epidermis and mucosa [52]; it comprises 4-10% of all malignant cancers and is correlated with 75% of skin cancer-related deaths [53]. The death rates associated with melanoma have reportedly dropped rapidly after the introduction of new therapies, including targeted therapies for melanoma metastasis and immune checkpoint inhibitors [54, 55]. Liu et al. [56] recently found that the regulation of miR-650 has a negative correlation with melanoma progression. Moreover, further study showed that miR-650 overexpression alleviated MGMT-induced DTIC resistance in melanoma by increasing cell apoptosis [19], which indicated that miR-650 was a novel biomarker of great value for the evaluation of melanoma.

### miR-650 and leukaemia

As the most frequent malignancy of the bone marrow, acute myeloid leukaemia (AML) has a high fatality rate [35]. Yuan et al. [57] revealed that miR-650 expression was reduced in AML, which contributed to leukaemia progression. Similarly, Gaine et al. [58] found that the expression of the erythropoietin receptor (EPOR) in t(12;21) B-ALL cells was higher than that in normal samples. Notably, EPOR expression is influenced by GATA2 and has an inverse correlation with miR-650 expression [58]. One study published in *Blood* reported that overexpression of miR-650 is correlated with a favourable chronic lymphocyte leukaemia (CLL) prognosis and affects the oncogenic capacity of B cells [24]. Furthermore, multivariate analysis showed that overexpression of miR-650 is an available independent predictor of survival and time to first treatment (TTFT) [24]. However, an inconsistent report that partly argued against the results above showed a significant increase in miR-650 expression in CLL patients [59]. Hence, the association between leukaemia and miR-650 needs further verification by expanding the number of patient samples included in the current work.

### Other tumours

miR-650 has also been found to serve as an oncogene that is upregulated in various other cancers. In breast cancer, overexpression of miR-650 has been observed to induce the downregulation of the tumour suppressor ING4, which we previously mentioned is significantly correlated with EMT of breast cancer cells [60]. Another recent study revealed that the expression level of miR-650 is upregulated in EC, resulting in the progression of EC through the SMAD7-TGF-β (transforming growth factor-β) pathway [11]. In addition, miR-650 overexpressed in anaplastic thyroid carcinoma (ATC), where it promotes the proliferation and motility of cancer cells by targeting Protein Phosphatase 2 Catalytic Subunit Alpha (PPP2CA) [61]. In osteosarcoma, miR-650 has been reported to function as an oncogene. Yun et al. [18] revealed that overexpressing miR-650 decreased ING4 expression in human osteosarcoma cells and increased IL-6 transcription. Both ING-4 and IL-6 could modulate osteoblast and osteoclast differentiation. Moreover, miR-650 could upregulate the transcriptional activity of NF κB [18] and reduce the quantity of nuclear factor (NF) of κ light polypeptide gene enhancer. Interestingly, similarly significant upregulation of miR-650 has been observed in oral cancer, and the expression of miR-650 has been positively correlated with cancer cell proliferation, migration and invasion [62]. Likewise, Zuo et al. [63] reported that miR-650 could function as an onco-miR in prostate cancer by suppressing cellular stress response (CSR1) expression.

Overall, these findings have revealed that the profile of miR-650 expression depends on the type of cancer and that miR-650 is involved in the occurrence and development of various cancers. The overall expression files of miR-650 in various cancers and relative clinical features are presented in Tables 1 and 2. Even so, more studies are required to further explore the expression profiles of miR-650 in tumours.

**Table 1:** Expression of miR-650 in various cancer and relative clinical significance

Cancer type	Expression	Samples	Clinical significance	References
HCC	Upregulated	Tissues from 130 HCC patients	Microscopic vascular invasion, tumour volume, TNM stage	[30]
HCC	Upregulated	/	/	[31]
HCC	Upregulated	248 HCC tissues and 120 paracarcinomatous HCC tissues	Age, differentiation capability, tumour stage	[34]
FLD/HCC	Upregulated	/	Tumour metastasis	[32]
NSCLC	Upregulated	53 NSCLC tissues and adjacent normal lung tissues	/	[64]
NSCLC	Upregulated	49 NSCLC tissues and adjacent normal tissues	Overall survival rate	[13]
NSCLC	Upregulated	/	/	[37]
LAD	Upregulated	96 LAD tissues and adjacent normal tissues, 44 LAD tissues (received docetaxel-based chemotherapy)	Lymph node metastasis, tumour stage, overall survival rate, docetaxel resistance	[16]
CRC	Downregulated	Tissues from 96 CRC patients	Overall survival rate	[39]
CRC	Upregulated	45 rectal cancer tissues, 22 colon cancer tissues, adjacent noncancerous tissues	Oxaliplatin resistance, tumour growth	[40]
CRC(bioinf)	Downregulated	Tissues from 217 CRC patients	/	[41]
CRC	Upregulated	Tissues from 70 CRC patients	Lymph node metastasis	[42]
CRC	Upregulated	/	/	[14]
GC(bioinf)	Upregulated	Tissues from 90 GC patients, tissues from 90 pre-GC patients, tissues from 45 healthy controls	/	[45]
GC	Upregulated	Tissues from 119 GC patients	Overall survival rate	[46]
GC	Upregulated	93 GC tissues and adjacent normal tissues, 44 LAD tissues (received docetaxel-based chemotherapy)	Tumor growth	[47]
GC	Upregulated	Primary GC tissues	Lymphatic and distant metastasis	[15]
Glioma	Upregulated	168 glioma tissues and 21 normal contral tissues	WHO grade, KPS score, overall survival rate	[49]
Glioma	Upregulated	Tissues from 39 glioma patients	Tumour formation and growth	[50]
Glioma	Downregulated	Tissues from 24 glioma patients	/	[51]
Melonoma	Downregulated	Tissues from 309 melonoma patients	TNM stage, overall survial, progression-free survival	[19]
Melonoma	Downregulated	/	/	[56]
CLL	Downregulated	Peripheral blood from 80 CLL patients and healthy controls	/	[24]
CLL	Upregulated	Peripheral blood from 102 CLL patients and 40 healthy controls	Binet stage, lactate dehydrogenase (LDH) level, time for first treatment	[59]
ALL	Downregulated	/	Prognosis	[58]
AML	Downregulated	Bone marrow and peripheral blood from 40 AML patients and 35 healthy controls	/	[57]
BC	Upregulated	Tissues from BC patients	/	[60]
EC	Upregulated	60 EC tissues and adjacent normal tissues	Tumour size, TNM stage, lymph and distant metastasis	[11]
ATC	Upregulated	12 PTC tissues, 5 ATC tissues, 6 normal tissues	/	[61]
Osteosarcoma	Upregulated	/	/	[18]
OC	Upregulated	/	Tumour weight and volume	[62]
PC	Upregulated	216 PC tissues, 324 benign prostate tissues, 77 normal tissues	Tumour volume, tumour metastasis, mortality of severe	[63]

**Table 2:** The roles of miR-650 in various cancer cell lines

Cancer type	Cell lines	Upstream	Target	Roles	Function	References
HCC	HCC cell and LO2	/	LAST2	Tumour promoter	Promotes cell proliferation, migration and invasion; increases cell EMT	[30]
HCC	LO2, SK-HEP-1, HUH-7, LM6 and Li-7	AXIN1	/	Tumour promoter	Promotes cell proliferation, migration and invasion; increases cell EMT	[31]
HCC	THLE-2 (CRL-2706, ATCC)	/	ING4	Tumour promoter	Promotes cell proliferation	[34]
FLD/HCC	SMMC-7721 and BEL-7404	Benzo[a]pyrene	SOCS3/JAK/STAT3	Tumour promoter	Promotes cell motility	[32]
NSCLC	H23, H522, A549, H1299, SPC-A1, 16HBE, HEK293T	/	LATS2	Tumour promoter	Promotes cell proliferation, migration and invasion	[64]
NSCLC	A549, NCI-H460, MRC-5	/	ING4	Tumour promoter	Promotes cell proliferation and invasion	[13]
NSCLC	cell line H1299, 293T	LncRNA MEG3	SLC34A2	Tumour promoter	Promotes cell migration and invasion; strengthens stem cell-like characteristics	[37]
LAD	SPC-A1, NCI-H1299	/	ING4/Bcl-2/Bax	Tumour promoter	Promotes cell growth; enhances the resistance of LAD cells to docetaxel; reduces the sensitivity of LAD cells to docetaxel	[16]
CRC	DLD-1, HCT-8, HEK293T	/	AKT2/GSK3β/E-cadherin pathway	Tumour suppressor	Suppresses cell proliferation, migration and invasion	[39]
CRC	Caco2 and HT29	LncRNA MIR155HG	ANXA2	Tumour promoter	Promotes M2 macrophage polarization and cell proliferation, migration, invasion and oxaliplatin resistance	[40]
CRC(bioinf)	/	NF-κB	NF-κB signalling pathway	Tumour suppressor	/	[41]
CRC	SW480, HT29, SW620, LOVO	/	NDRG2	Tumour promoter	Promotes cell growth; suppresses cell differentiation and apoptosis	[42]
CRC	SW480, SW620, RKO, 320DM, 320HSR, NCI-H716, H508, CCD841CoN	/	RhoA/Rac1 GTPase + ING4/ERK/p38 MAPK	Tumour promoter	Promotes cell proliferation and migration; increases cell EMT	[14]
GC(bioinf)	/	/	/	Tumour promoter	/	[45]
GC	HGC-27, MKN-45 cells, HEK293T	PBX1	LATS2	Tumour promoter	Promotes cell proliferation, invasion and migration; suppresses cell apoptosis	[46]
GC	AGS, HGC-27, MGC-803, SGC-7901, GES-1	LncRNA DICER-AS1	CSR1	Tumour promoter	Promotes cell proliferation, migration; suppresses cell apoptosis	[47]
GC	SNU-16, NCI-N87	/	ING4	Tumour promoter	Promotes cell proliferation and tumorigenesis	[15]
Glioma	/	/	/	Tumour promoter	/	[49]
Glioma	U251, LN229, U373, A172, U87, NHA	NF-κB	RERG-PHLPP2/AKT + ERK + NF-κB	Tumour promoter	Promotes cell proliferation, autophagy, migration and invasion; increases EMT	[50]
Glioma	LN229, U87, U251, LN308, SNB19, H4	/	FAM83F	Tumour suppessor	Promotes cell proliferation	[51]
Melonoma	A375, MV3	LncRNA POU3F3	/	Tumour suppessor	Reduces MGMT-induced DTIC resistance; suppress cell proliferation	[19]
Melonoma	M21, SK-MEL-1, A2058, A375, HEMa-LP	LncRNA ZFPM2-AS1	Notch1	Tumour suppessor	Promotes cell proliferation and migration; suppresses cell apoptosis	[56]
CLL	NALM-6	host gene for IgLλ	CDK1 ING4 EBF3	Tumour suppessor	Suppresses proliferative capacity of B cells	[24]
CLL	CLL cells	/	NDRG2	Tumour promoter	Suppresses cell apoptosis	[59]
ALL	REH, NALM-6, UT-7	/	GATA2/EPOR	Tumour suppressor	/	[58]
AML	K562 cells	/	Gfi1	Tumour suppressor	Suppresses cell proliferation	[57]
BC	UIVC-IDC-6, -9, -10	22q11.2 gene	ING4 NDRG2	Tumour promoter	Increases cell EMT	[60]
EC	HEC-1B, HEC-1 A, Ishikawa, RL-952, hEEC	LncRNA MCTP1-AS1	SMAD7/TGF-β/SMAD pathway	Tumour promoter	Promotes cell proliferation, migration and invasion; increases EMT	[11]
ATC	TPC-1, CAL62, SW1736, 8505 C	/	PPP2CA	Tumour promoter	Promotes cell migration and invasion	[61]
Osteosarcoma	MG-63	/	NFκB + ING4/IL-6	Tumour promoter	/	[18]
OC	hTERT-OME, SCC-15, SCC-4, SCC-9, SCC-25, CAL-27, FaDu, 019	/	Gfi1	Tumour promoter	Promotes cell proliferation, migration and invasion	[62]
PC	PC3, DU145	/	CSR1	Tumour promoter	Promotes cell growth and DNA replication	[63]

## Functions of miR-650 and the related mechanisms

**Fig. 1:**
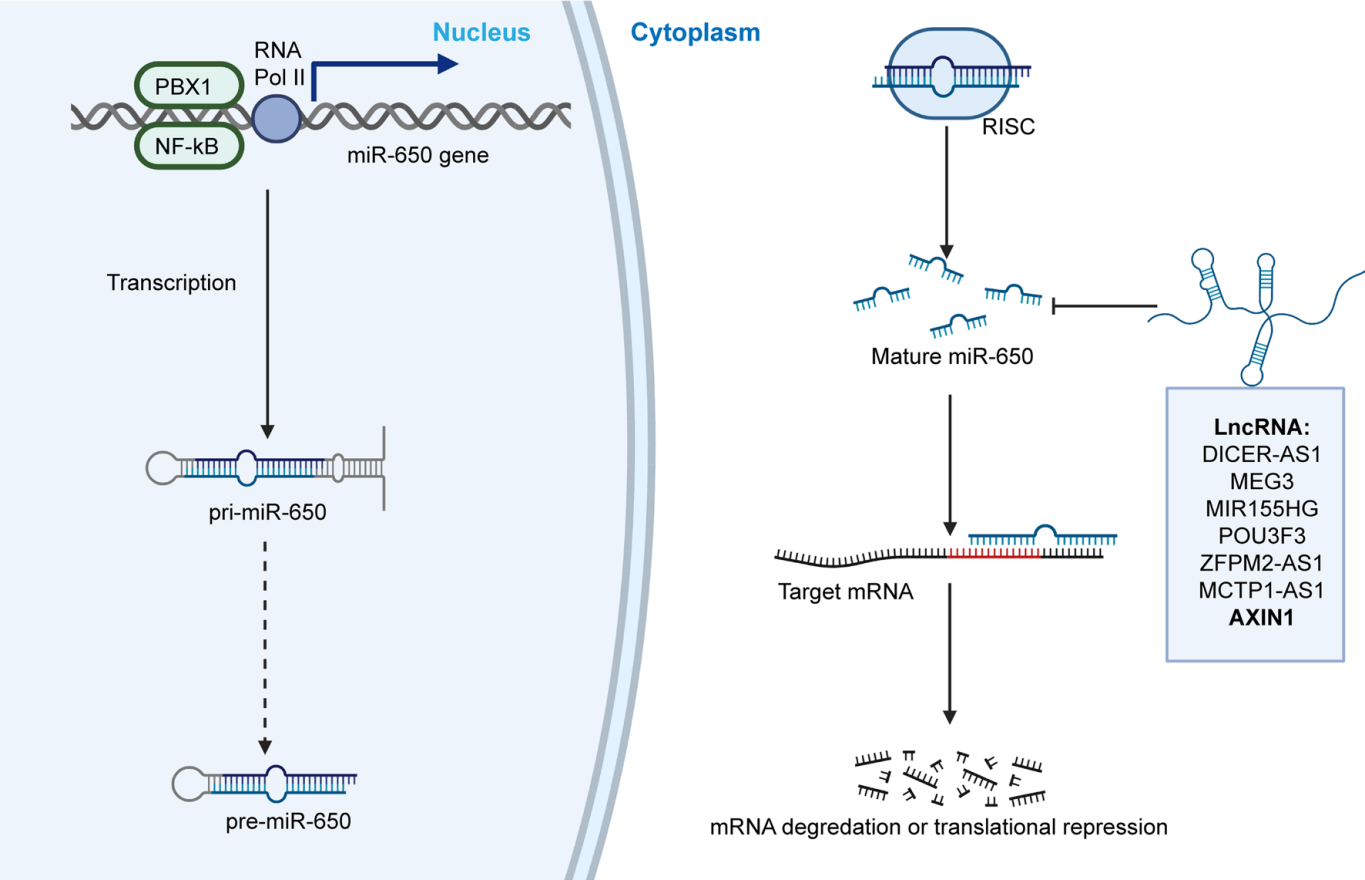
Upstream regulation of miR-650. Multiple factors participate in the upstream regulation of the miR-650 biological process. In the nucleus, transcription of miR-650 is modulated by coupling expression with its homologous gene immunoglobulin lambda and promoted by transcription factors PBX1 and NF-kB. In the cytoplasm, AXIN1 and lncRNAs (DICER-AS1, MEG3, MIR155HG, POU3F3, ZFPM2-AS1, MCTP1-AS1), which account for the major alterations, act as molecular sponges for miR-650 and negatively regulate its biological function, inducing cancer gene deregulation

**Fig. 2:**
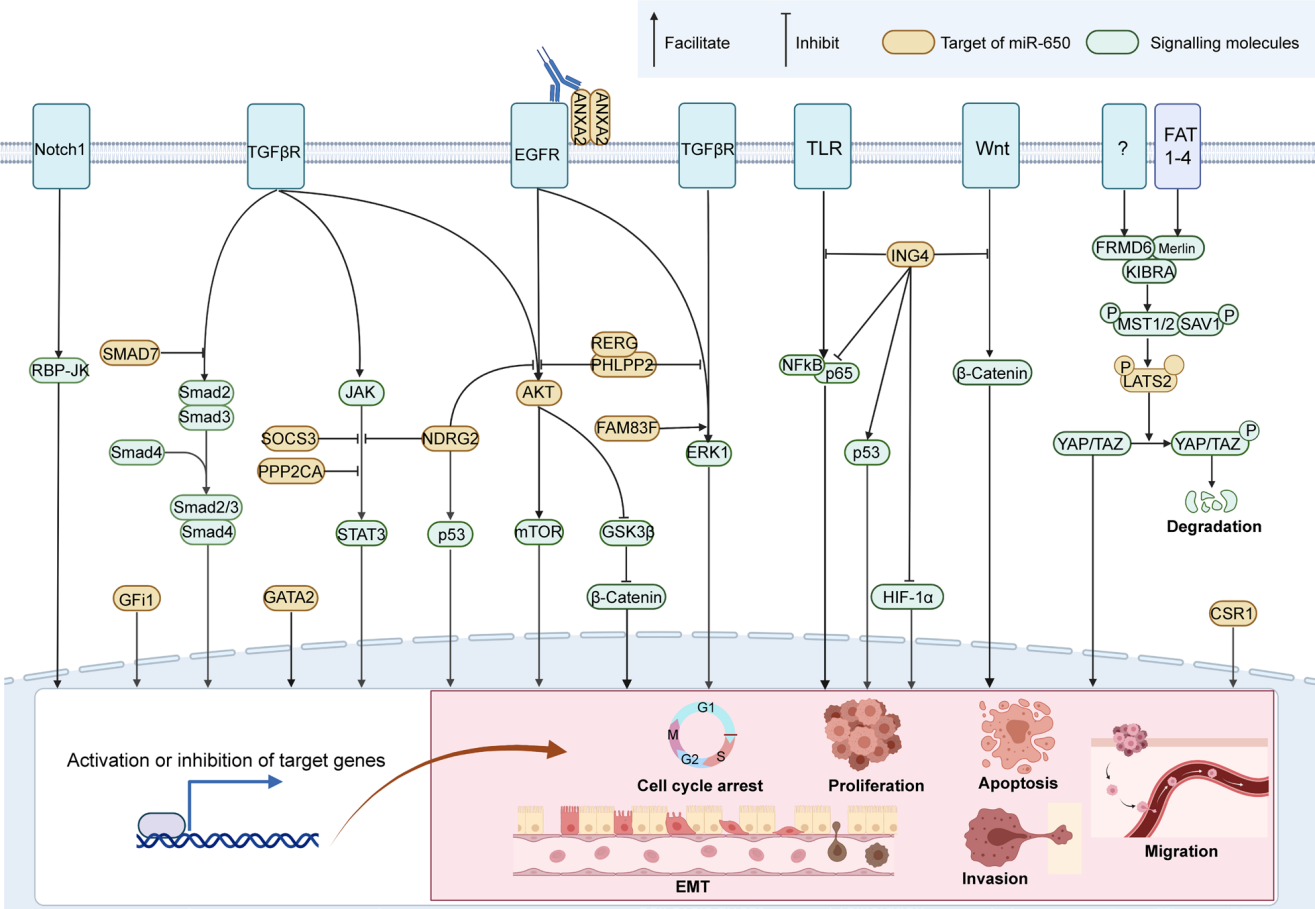
Target regulators of miR-650, related signalling pathways and induced biological effects. Genesis, differentiation and metastasis of tumours are essentially related to diverse intracellular signalling pathways, such as Jagged/Notch1, TGFβ/SMAD, Wnt/β-Catenin, and FAT/LATS2, which deliver multiple signals to the cell nucleus in a cascade mode and then trigger a series of biological effects. miR-650 functions as a tumour promoter or suppressor by posttranscriptionally regulating the pivotal factors (oncogenic or anti-oncogenic) within these cascades to affect various biological processes, including cell cycle arrest, proliferation, apoptosis, migration, invasion and EMT. The integrated signalling pathways and key factors directly targeted by miR-650 (white blue) are shown

In addition to the abberant expression files of miR-650, researchers have also observed variations in the biological functions of miR-650 through in vitro and in vivo experiments. Generally, miR-650 functions as an onco-miR or tumour suppressor by affecting biological processes such as cell proliferation and apoptosis. In the next section, we summarize the dual roles of miR-650 in tumours and the signalling pathways involved. Additionally, the comprehensive functions and related mechanisms of miR-650 in various tumours are presented in Figs. 1 and 2.

### miR-650 functions as a tumour promoter

In parallel with the overexpression and clinical features discussed above, miR-650 also conferred more oncogenicity to tumour cells. In HCC, existing evidence indicates that upregulation of miR-650 modulates cell proliferation, apoptosis, migration and invasion [30–32, 34] of cancer cells. In terms of the mechanism, miR-650 functions as an upstream signal of the large tumor suppressor kinase 2 gene (LATS2)/YAP (Ser127) signalling pathway and regulates its downstream target genes [30]. Moreover, Ye et al. [64] also revealed that miR-650 could serve as an onco-miR to potentiate cell growth and metastasis by directly targeting LATS2 in NSCLC formation and progression. Interestingly, Liu et al. [46] reported that miR-650 downregulation inhibited proliferation, expedited apoptosis and reduced the migration of HP + GC cells. Moreover, miR-650 mediated the Hippo pathway via the PBX1/miR-650/LATS axis. As a molecular sponge of Axin1, miR-650 enhanced the Wnt signalling pathway to facilitate tumour progression [31]. Furthermore, Ge et al. [32] found that miR-650 in benzo[a]pyrene-exposed cancer cells contributed to HCC metastasis by directly targeting suppressor of cytokine signalling 3 (SOCS3), and this inhibition modulated the activation of the Janus kinase (JAK)/ signal transducer and activator of transcription 3(STAT3) cascade. The study from Zhao et al. [37] suggested that miR-650 silencing inhibited the vital capacity and invasion ability of large-cell carcinomas (LCCs) and lung cancer stem cells (LCSCs) (H1299 cell lines) through the lncRNA maternally expressed gene 3(MEG3)/miR-650/solute carrier family 34 member 2 (SLC34A2) axis. Moreover, ING4 is also a vital gene targeted by miR-650 to facilitate cell proliferation and invasion and induce a stem cell-like state in LC [13, 16]. With regard to CRC, Zhou et al. [40] argued that MIR155HG, as an endogenous lncRNA, competed with annexin 2 (ANXA2) by combining with miR-650, thereby promoting M2 macrophage polarization and oxaliplatin resistance in CRC cells. In another study, decreased luciferase activity of miR-650 was observed with the 3’ untranslated region of N-myc downstream regulated gene 2 (NDRG2) inserted downstream of the luciferase gene, indicating that NDRG2 was directly targeted by miR-650 [65]. Notably, other researchers have observed that inhibition of miR-650 facilitates more apoptosis in CLL cells [59]. Further studies have demonstrated that NDRG2 expression can be significantly downregulated by miR-650 simultaneously with p53 aberrations [59]. It is worth mentioning that other researchers have found that constructed adenoviruses carrying the NDRG2 gene heightened p53-mediated apoptosis of HCC cells [66]. Moreover, CSR1 has also been reported to be a shared gene targeted by miR-650 in both GC [47] and prostate cancer [63]. In addition to cell proliferation and apoptosis, overexpression of miR-650 induces EMT of cancer cells [11, 14, 30, 49, 67], which is a key factor in promoting the metastasis of tumours [68]. Functional analyses in Jin’s study demonstrated that upregulated miR-650 expression heightened the migration of glioma cells through EMT promotion. Additionally, they found that miR-650 could inhibit glioma cell adhesion and promote autophagy. Mechanistically, NF-κB1 upregulated miR-650 expression by directly interacting with its promoter, and then the AKT/ extracellular regulated protein kinases (ERK) and NF-κB pathways were enhanced by miR-650 via the RAS-like, estrogen-regulated growth inhibitor (RERG)-PH domain and leucine rich repeat protein phosphatase 2 (PHLPP2) complex [50].

As a valued member of the ING family, ING4 has been revealed to function as a formidable tumour suppressor due to its significant role in the modification of chromatin modification, cell growth, cell invasion and vascularization [69, 70]. However, it has been revealed to be frequently decreased in various human tumours, and the variation in ING4 markedly contributes to cancer development. Interestingly, accumulating studies have suggested that ING4 is a downstream target of miR-650 in many types of cancer, such as HCC [34], LC [13, 16], CRC [14], GC [15], and BC [60]. Finally, You et al. [14] demonstrated that miR-650 could function as a tumour promoter and enhance the malignant phenotype of CRC. In terms of the mechanism, miR-650 targets ING4, leading to CRC progression promoted by the ERK/p38 mitogen-activated protein kinases (MAPK) pathway. Analogously, another study further suggested that miR-650 increased caspase-3-dependent cell apoptosis by regulating Bcl-2/Bax expression [16] via ING4. Interestingly, Tang et al. [13] revealed that miR-650 promotes NSCLC cell proliferation and migration through the ING4/Wnt-1/β-catenin pathway. Combined, these results suggest that ING4 is a significant regulator of the signalling pathways in the tumorigenic progress of miR-650 and provides promising biomarker and therapeutic target for human cancer.

### miR-650 functions as a tumour suppressor

In contrast with the aforementioned investigations, miR-650 has also been reported to function as a tumour suppressor by arresting the cell cycle, inhibiting cell proliferation and weakening the malignant phenotype in tumours. In CRC, there is no consensus about the function of miR-650 thus far. Zhou et al. [39] found that miR-650 inhibited cell growth and migration by suppressing the AKT2/ Glycogen synthase kinase (GSK3β)/E-cadherin pathway. Another bioinformatic study revealed a similar conclusion [41, 42]. Therefore, to further explore the roles of miR-650, more related studies are needed. In addition, rescue experiments in Xu’s et al. [51] study showed that miR-650 could inhibit cell growth by targeting family with sequence similarity 83member F (FAM83F) in glioma. In addition, the lncRNA POU3F3/miR-650/MGMT pathway has been revealed to function critically in DTIC resistance in melanoma [19]. In terms of the mechanism, lncRNA POU3F3 works as a competitive RNA to combine with miR-650; therefore, MGMT expression rises to a higher extent [19] in melanoma cells. Additionally, Liu et al. [56] revealed that miR-650 contained cell proliferation and invasion while exerting on adverse effect on cell apoptosis in cutaneous melanoma via the lncRNA ZFPM2-AS1/miR-650/NOTCH1 axis in melanoma. An article published in *Blood* indicated that overexpression of miR-650 led to the decreased proliferative capacity of B cells [24]. Mechanistically, miR-650 targeted essential proteins in cell proliferation, namely, cyclin-dependent kinase (CDK1), ING4, and EBF3 in B cells. A cell transfection experiment with miR-650 revealed significant downregulation of signalling molecule levels of EBF3, ING4 and CDK1 by 67%, 64%, and 53%, respectively. This finding validates the correlation of these three proteins with miR-650 in B cells. Moreover, Mraz et al. [24] found that miR-650 was modulated by coupling expression with its homologous gene for immunoglobulin lambda. This observation is surprising because previous studies demonstrated that miR-650 has an independent expression of immunoglobulin [23].

## Conclusions

In this review, we discussed the aberrant expression profiles, functions and underlying mechanisms of miR-650 in various cancer tissues and cell lines and focused on its upstream regulators and downstream target genes. As an important member of the miRNA family, miR-650 has been identified to play crucial roles in cancer genesis and progression via diversified signalling molecules, such as ING4, Gfi1, and LATS2, thereby regulating the proliferation, apoptosis, invasion and migration, EMT, and drug resistance of cancer cells. Taken together, accumulating evidence at present suggests that miR-650 can function as a tumour promoter in HCC, LC, GC, BC, EC, ATC, OC, PC, as a tumour suppressor in melanoma, and as both in CRC, glioma, and leukaemia. The discrepancy of its roles in cancer indicates that the functions of miR-650 are influenced by multiple factors, such as cancer cell types and the microecological environment. To fully understand its expressional functions, further studies of miR-650 molecular mechanisms are required.

This overview of the signalling pathways influenced by miR-650 provides us with a more comprehensive understanding of the complex association between miR-650 and human cancer. More significantly, valuable clues regarding the implications of miR-650 based on a constructed lncRNA-miRNA-mRNA molecular network will bring us more effective diagnostic and/or therapeutic strategies for various cancer patients. When arguing the suitability of this miRNA as a biomarker or therapeutic target, researchers need to discuss its availability and detectability in bodily fluid or exosomes and assess the stability in clinical tests.

## Data Availability

Not applicable.

## Abbreviations

miRNAs: microRNAs
KRAS: Kirsten rat sarcoma viral oncogene homologue
PD-1: Programmed cell death protein 1
EC: Endometrial cancer
EMT: Epithelial to mesenchymal transition
ING4: Inhibitor of growth 4
HCC: Hepatocellular carcinoma
NASH: Non-alcoholic steatohepatitis
LC: Lung cancer
NSCLC: Non-small-cell lung cancer
LAD: Lung adenocarcinoma
CRC: Colorectal cancer
GC: Gastric cancer
AML: Acute myeloid leukaemia
EPOR: Erythropoietin receptor
CLL: Chronic lymphocyte leukaemia
TTFT: Time to first treatment
ATC: Anaplastic thyroid carcinoma
NF: Nuclear factor
SOCS3: Suppressor of cytokine signalling 3
LCCs: Large-cell carcinomas
LCSCs: Lung cancer stem cells
AXIN1: Axis inhibition protein 1
CDKN1B: Cyclin-dependent inhibitor 1B
DTIC: Dacarbazine
MGMT: Methylguanine-DNA-methyltransferase
TGF-β: Transforming growth factor-β
PPP2CA: Protein Phosphatase 2 Catalytic Subunit Alpha
CSR1: Cellular stress response
LATS2: Large tumor suppressor kinase 2 gene
JAK: Janus kinase
STAT3: Signal transducer and activator of transcription 3
MEG3: Maternally expressed gene 3
SLC34A2: Solute carrier family 34 member 2
ANXA2: Annexin 2
NDRG2: N-myc downstream regulated gene 2
ERK: Extracellular regulated protein kinases
RERG: Estrogen-regulated growth inhibitor
PHLPP2: pH domain and leucine rich repeat protein phosphatase 2
MAPK: Mitogen-activated protein kinases
GSK3β: Glycogen synthase kinase 3β
FAM83F: Family with sequence similarity 83member F
CDK1: Cyclin-dependent kinase
